# Supplementary material for: Antimycobacterial Activities of Hydroxamic Acids and Their Iron(II/III), Nickel(II), Copper(II) and Zinc(II) Complexes
Source: Microorganisms. 2023 Oct 23;11(10):2611. doi: 10.3390/microorganisms11102611 (PMC10609363; doi:10.3390/microorganisms11102611)
Supplement: Supplementary file 1 [file microorganisms-11-02611-s001.zip › microorganisms-2610359-supplementary.pdf]

Supplementary Figure

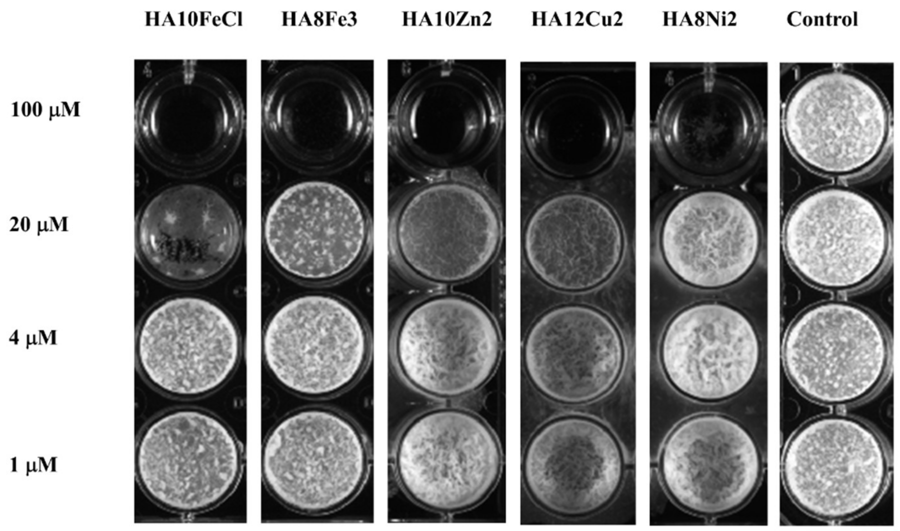

Figure S1. Representative *M. bovis* BCG biofilm formation assay. The *M. bovis* BCG was grown in Sauton's medium in the presence or absence (control) of various compounds (1-100 μM) for 24 days.

Supplementary Table

Table S1. Differential protein amounts identified by comparing HA10FeCl treated and untreated *M. bovis* BCG.

Index	Protein name	Gene name	Molecular Weight (kDa)	LFQ intensity		Fold change HA10FeCl /BCG	t test *p* value
				HA10FeCl	BCG		
1	HTH tetR-type domain-containing protein	*BCG_2177c*	21.614	14520.95	164180	0.0884	0.0002
2	Conserved hypothetical proline and threonine rich protein	*BCG_0352*	64.129	765540	634273.3	1.207	0.0032
3	UPF0353 protein BCG_1543	*BCG_1543*	36.03	12012667	12857333	0.9343	0.0032
4	Transcriptional activator protein	*BCG_2384c*	48.381	1981267	3505300	0.5652	0.0034
5	tRNA threonylcarbamoyladenosine biosynthesis protein TsaE	*BCG_3492c*	18.214	1522100	1278200	1.1908	0.0039
6	Probable methylmalonyl-COA mutase large subunit mutB	*mutB (BCG_1556)*	80.603	17600000	16619667	1.059	0.0042
7	DNA topoisomerase 1	*topA (BCG_3704c)*	102.37	44207667	39517000	1.1187	0.0044
8	Cyclic pyranopterin monophosphate synthase	*moaC3 (BCG_3390c)*	18.555	950413.3	783746.7	1.2127	0.0047
9	Large ribosomal subunit protein uL2	*rplB (BCG_0754)*	30.576	74363000	68214000	1.0901	0.0054
10	Indole-3-glycerol phosphate synthase	*trpC (BCG_1649)*	28.023	34303000	32548333	1.0539	0.0057
11	NADH-dependent glutamate synthase (Small subunit) gltD	*gltD (BCG_3921c)*	53.401	18078000	15521000	1.1647	0.0059
12	Thiazole synthase	*thiG (BCG_0456)*	25.894	865536.7	1122833	0.7709	0.0071
13	NADH-quinone oxidoreductase subunit A	*nuoA (BCG_3168)*	13.975	4408700	4133400	1.0666	0.0072
14	Probable integral membrane protein	*BCG_3545c*	23.793	607810	509986.7	1.1918	0.01
15	Probable transcriptional regulatory protein (Probably tetR-family)	*BCG_1076*	21.701	1806133	2037933	0.8863	0.01
16	Small ribosomal subunit protein bS6	*rpsF (BCG_0084)*	10.935	39701000	44250333	0.8972	0.0101
17	Calcineurin-like phosphoesterase domain-containing protein	*BCG_2813c*	37.6	5039900	4367967	1.1538	0.0114
18	Probable polyketide synthase pks12	*pks12 (BCG_2067c)*	431.54	78558333	71357333	1.1009	0.0127
19	Possible transcriptional regulatory protein (Probably tetR-family)	*BCG_0702c*	24.727	715100	694466.7	1.0297	0.0134
20	Rubredoxin	*BCG_3279c*	6.7974	70134.5	31983	2.1929	0.0141
21	Phosphate transport system permease protein	*pstC2(BCG_0981)*	34.261	87300.33	40007.33	2.1821	0.0143
22	Probable mutator protein mutT2	*mutT2 (BCG_1222)*	15.16	1039500	922426.7	1.1269	0.0157
23	DUF427 domain-containing protein	*BCG_1708*	13.11	18771.5	15052.67	1.2471	0.0158
24	Ribonuclease P protein component	*rnpA_2 (BCG_3981c)*	13.942	1077833	976606.7	1.1037	0.0162
25	Probable cytochrome P450 124 CYP124	*cyp124 (BCG_2283)*	47.824	90852.33	262460	0.3462	0.0162
26	Possible adenylate cyclase	*BCG_1381c*	61.934	3759367	4121333	0.9122	0.0167
27	Large ribosomal subunit protein uL4	*rplD (BCG_0752)*	23.743	28623667	26318000	1.0876	0.0182

28	ESX-1 secretion-associated protein EspA/EspE-like domain-containing protein	*BCG_3927*	43.506	11706667	10565333	1.108	0.0199
29	thiosulfate sulfurtransferase	*BCG_0364*	24.091	809230	652393.3	1.2404	0.0205
30	Signal transduction histidine-protein kinase/phosphatase MprB	*mprB (BCG_1037)*	54.431	3165333	3524033	0.8982	0.0213
31	Uncharacterized protein	*BCG_2826*	9.8619	776093.3	648636.7	1.1965	0.0216
32	50S ribosomal protein L25	*rplY (BCG_1072c)*	22.441	28694667	26092000	1.0997	0.022
33	DUF222 domain-containing protein	*BCG_0128c*	29.034	234963.3	188753.3	1.2448	0.023
34	Putative S-adenosyl-L-methionine-dependent methyltransferase BCG_1935c	*BCG_1935c*	33.267	6691433	5960633	1.1226	0.0231
35	Probable oxidoreductase	*BCG_2793c*	33.517	304546.7	556953.3	0.5468	0.0237
36	Probable lipase/esterase lipG	*lipG (BCG_0695c)*	32.963	6949833	5992800	1.1597	0.0242
37	Probable beta-glucosidase bglS	*bglS (BCG_0223)*	73.552	669126.7	449480	1.4887	0.0249
38	Putative phthiocerol dimycocerosate transporter LppX	*lppX (BCG_2967c)*	24.14	55880000	53324000	1.0479	0.0268
39	Probable propionyl-CoA carboxylase beta chain 4 accD4	*accD4 (BCG_3861c)*	56.679	25602667	24137667	1.0607	0.0275
40	Transmembrane protein	*BCG_1127c*	64.357	6147900	5556067	1.1065	0.0293
41	tRNA pseudouridine synthase A	*truA (BCG_3520c)*	28.092	6145633	5449833	1.1277	0.0295
42	Thioredoxin	*trxC_1 (BCG_0020)*	12.544	16445000	17423000	0.9439	0.0301
43	Possible conserved membrane protein	*BCG_3932*	51.092	1164233	891630	1.3057	0.0306
44	Cell division protein CrgA	*crgA (BCG_0011c)*	10.43	928696.7	605450	1.5339	0.0327
45	Probable transmembrane protein	*BCG_1629*	23.183	2499733	2330567	1.0726	0.0331
46	Probable conserved transmembrane alanine rich protein	*BCG_2863*	17.767	810793.3	931126.7	0.8708	0.0348
47	Endolytic murein transglycosylase	*mltG (BCG_2576c)*	45.473	6583633	5878633	1.1199	0.0357
48	Glutamyl-tRNA amidotransferase	*BCG_3747c*	16.701	3316733	3643000	0.9104	0.0364
49	VOC domain-containing protein	*BCG_1384c*	16.626	1672967	1359033	1.231	0.0367
50	Probable conserved transmembrane transport protein mmpL4	*mmpL4 (BCG_0489c)*	105.23	9818967	9134000	1.075	0.0376
51	Small ribosomal subunit protein uS4	*rpsD (BCG_3523c)*	23.476	110000000	104000000	1.0604	0.0393
52	Possible conserved membrane protein	*BCG_1478*	16.383	1205787	623100	1.9351	0.0395
53	Phthiotriol/phenolphthiotriol dimycocerosates methyltransferase	*BCG_2973*	30.651	39149667	34174333	1.1456	0.0397
54	Dolichyl-phosphate-mannose--protein mannosyltransferase	*BCG_1059c*	55.544	749520	587350	1.2761	0.0401
55	Possible conserved membrane protein	*BCG_0582*	55.016	4408433	4119933	1.07	0.0457
56	Probable oxidoreductase	*BCG_0099*	32.413	4390933	3837100	1.1443	0.0469
57	Glycosyltransferase 2-like domain-containing protein	*BCG_1268*	34.379	1285367	1138700	1.1288	0.0473
58	Probable conserved membrane protein mmpS4	*mmpS4 (BCG_0490c)*	15.403	2704100	2379033	1.1366	0.0479

LFQ: protein quantification; HA10FeCl: HA10FeCl-treated *M. bovis* BCG.

Table S2. Differential protein amounts unidentified by comparing HA10FeCl treated and untreated *M. bovis* BCG.

**Index**	**Protein name**	**Gene name**	**Molecular Weight (kDa)**	**LFQ intensity**	
				**Average HA10FeCl**	**Average BCG**
1	IS like-2 transposase	*BCG_1100c*	15.189	69579	NA
2	Possible nitrate/nitrite transporter narK2	*narK2* (*BCG_1776c*)	41.109	2638.747	NA
3	Probable aldehyde dehydrogenase	*BCG_0259c*	28.491	81704.5	NA
4	DUF732 domain-containing protein	*BCG_3092*	13.915	46762	NA
5	PE family protein	*PE15* (*BCG_1447*)	9.862	NA	1619
6	Probable conserved transmembrane transport protein mmpL9a	*mmpL9a* (*BCG_2361*)	89.958	NA	169034.3

LFQ: protein quantification; HA10FeCl: HA10FeCl-treated *M. bovis* BCG; NA: unidentified with lower expression.
